# Emerging Trends and New Developments in Transient Elastography: A Bibliometric and Cocitation Analysis from 1999 to 2017

**DOI:** 10.1155/2019/3280657

**Published:** 2019-03-05

**Authors:** Jingjing Gu, Weimian Wang, Ziyuan Zou, Fangze Huang, Chihua Fang, Xun Li, Xiaolong Qi

**Affiliations:** ^1^CHESS Frontier Center Working Party, The First Hospital of Lanzhou University, Lanzhou University, Lanzhou, China; ^2^CHESS Group, Hepatic Hemodynamic Lab, Institute of Hepatology, Nanfang Hospital, Southern Medical University, Guangzhou, China; ^3^CHESS Group, The First Department of Hepatobiliary Surgery, Zhujiang Hospital, Southern Medical University, Guangzhou, China; ^4^CHESS Frontier Center, Lanzhou University, Lanzhou, China

## Abstract

**Purpose:**

To identify and characterize the 100 most-cited articles in the field of transient elastography.

**Methods:**

The top-cited articles focusing on transient elastography from 1999 to 2017 were retrieved from Science Citation Index Expanded (SCI-E) database. The most prolific article, journal, country and continent, top-cited article in different period, international collaboration, cocitation analysis of journal were retrieved and analyzed in this article. Bibexcel 2016, Microsoft Excel 2010, and VOSviewer 1.6.5 were used to analyze bibliometric records we downloaded.

**Results:**

The 100 most-cited articles were published between 2003 and 2015. The total citations ranged from 54 to 1376 (mean, 167.52 ± 208.46; median, 89.5) and the annual citations ranged from 4.91 to 98.24 (mean, 17.21±15.68; median, 12.1). The top-cited article was published in* Gastroenterology* in 2005 by Castera L. et al. (n=1376). The most-cited articles in 2003-2007 (n=1380), 2008-2012 (n=599) and 2013-2017 (n=159) were located. All of the most-cited articles in three periods were focusing on the topic of noninvasive assessment of liver fibrosis. The most prolific author was de Ledinghen V. (n=21) and France (n=43) was the leading country. The most productive journal was* Journal of Hepatology* (n=20). The major article type was original research article.

**Conclusions:**

We undertook efforts to provide an insight into the features and evolvement of the most-cited articles in the field of transient elastography. For transient elastography, as a noninvasive assessment of liver fibrosis, its use in the evaluation of liver fibrosis is gradually mature and shows great advantages. Moreover, the field of transient elastography is in a stage of rapid development.

## 1. Introduction 

Transient elastography is a reproducible, reliable, and noninvasive technique for assessing liver fibrosis in patients with chronic liver disease [1], which provide an estimate of the liver elasticity by measuring the propagation speed of shave wave [2]. Since the first publication on transient elastography in 1999, the development of transient elastography has flourished which could be manifested by numerous relevant articles published in science journals. Bibliometric analysis not only is a method to attain the trends and conditions of a specific research field, but also can affect the progress of it in the future [3]. The rate at which article is cited or referenced by other researchers is used to determine the importance of a science article in a specific field [4–8], since it indicates the influence of previous work on subsequent researches. Although the citation analysis with intrinsic limitations is not an accurate measurement for a research's quality, this method has become a popular method of estimating academic performance, which is associated with the influence of the articles in practice, discussion, and further research [4, 5, 9, 10]. With these advantages, the citation analysis has been carried out in various medical subspecialties, such as hypertension [11], diabetes [12], digestive system diseases [13], respiratory system diseases [14], obstetric and gynecological diseases [15], integrative and complementary medicine [16], and medical imaging [17].

However, as far as is known, study of the most-cited article in transient elastography is still absent. Herein, with an objective to provide a more in-depth understanding of evolution of the burgeoning, noninvasive diagnostic method for examining the liver cirrhosis, we conducted this study to identify and characterize the 100 most-cited articles in the field of transient elastography.

## 2. Methods

### 2.1. Search Strategy

The first step of bibliometric analysis is retrieving and analyzing the literature on a certain topic. In this study, the Science Citation Index Expanded (SCI-E) was used to identify the 100 most-cited articles. Transient elastography was used as the search term. Considering transient elastography was invented in 1999, we set a large time span from 1999 to 2017, aiming to cover all the potential articles.

### 2.2. Evaluating the Included Studies

One may question the validity of using transient elastography as the search term without any limitation. In May 2018, all article identification was independently conducted by two reviewers (JG and WW), regardless of article type and language. Any disagreement between reviewers was resolved by a third reviewer (XQ). Included criterion was that transient elastography was applied as the only or one of the methods to measure liver stiffness.

### 2.3. Assessing the Articles and Journals

The following information was extracted from the 100 articles: (1) publication year; (2) overall citations; (3) number of annual citations (overall citations/year after publication); (4) journal title; (5) journal category; (6) impact factor of the journal (IF based on the 2017 science edition of the ISI JCR); (7) the citation received by the publication; (8) authorship; (9) country of articles and international collaboration.

Statistical analysis Bibexcel (version 2016-02-20) was used to extract information from downloaded bibliometric records and Microsoft Excel 2010 program was used to accomplish most of data presented in this study. Statistical analysis in this study was performed by using SPSS 17 (SPSS Inc., Chicago, IL, USA). Pearson correlation coefficient was used to examine citation times and annual citation times with an alpha level of 0.05. The visualization of international collaboration and cocitation analysis of journal was accomplished with the use of VOSviewer (version 1.6.5).

Our study was a bibliometric analysis and an ethic committee approval was not required in this research.

## 3. Results

**Figure 1:**
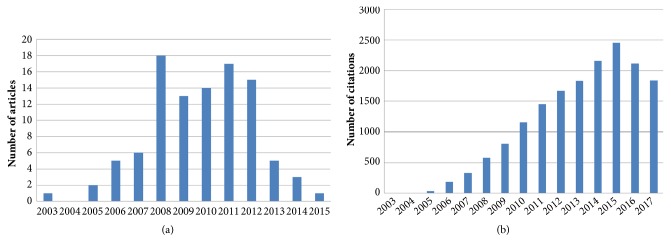
Developing trends in research on transient elastography. (a) The number of most-cited articles in each year. (b) The number of article citations in each year. If no article was in the list of the 100 most-cited articles in a certain year, there will be no bar shown in that year.

Included articles in this study were published between 2003 and 2015. The total citations of the 100 most-cited article in the field of transient elastography ranged from 54 to 1376 (mean, 167.52  ±208.46; median, 89.5). The annual citations ranged from 4.91 to 98.29 (mean, 17.21±15.68; median, 12.1). The article with both the highest total citations and annual citations was published in* Gastroenterology* in 2005 by Castera L. et al. (1376). The details of total citations and annual citations were presented in Table S1. The numbers of most-cited articles and citations in each year were presented in Figures 1(a) and 1(b), respectively.

**Figure 2:**
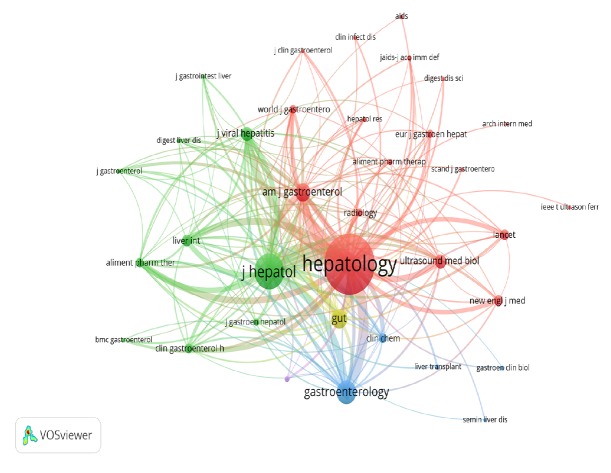
Journal cocitation analysis. Journals with a minimum of 20 citations were included. The size of the circles represents the total link strength of the journal, and the width of line shows the link strength between two journals. Only the link strength more than 100 will be displayed. The different colors represent different clusters.

The 100 articles were published in 33 journals and the impact factor of the journal ranged from 12.92 to 18.39. The journal containing the most included articles in the study was* Journal of Hepatology* (n=20), followed by* Hepatology* (n=14) and* Liver International* (n=7). Journals with more than three articles in the 100 most-cited articles were presented in Table 1. Cocitation analysis of the most productive journals in the topic of transient elastography was presented in Figure 2. Journals with a minimum of 20 citations were included. The total link strength of the journal was represented by the circle size and the width of the line indicated the link strength between two journals (the number of cocitations). Different colors represented different clusters. The journals in the same cluster focused on a similar research field.* Hepatology* was commonly cocited with* Journal of Hepatology* (link strength=9051),* Gastroenterology* (link strength=4110),* American Journal of Gastroenterology *(link strength=3232),* Gut *(link strength=3040),* Journal of Viral Hepatitis* (link strength=2470), and* Liver International *(link strength=2047).

**Figure 3:**
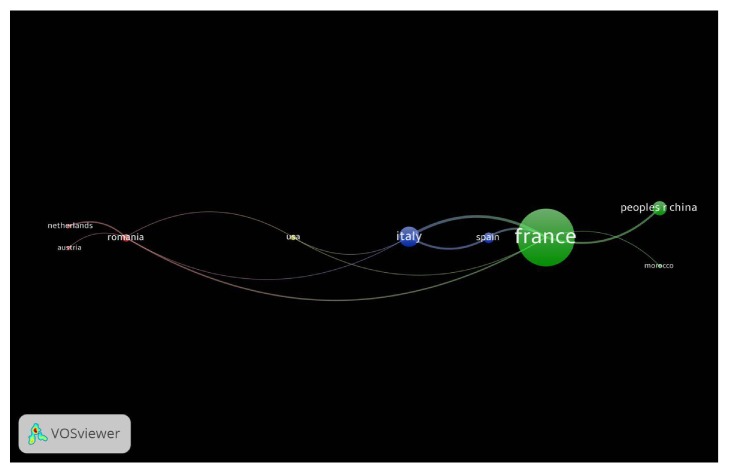
Visualization of international collaboration between countries; the size of the circles represents the collaboration degree, and the width of line illustrates the strength. If a country did not cooperate with other countries to accomplish one of the 100 cited-most articles, it will not be shown in the figure.

The most prolific author in this field was de Ledinghen V. (n=24), followed by Castera L. (n=17), and Foucher J. (n=15), all of whom were from France.  Table 2 showed a list of authors who had published more than five included articles. The 100 most-cited articles were contributed by 16 different countries. As for the included publication times, France (n=43) was the leading country while Italy (n=15) and China (n=11) ranked the second and the third, respectively. The international collaboration (if an article is completed by authors from different countries, it is regarded as an international collaboration) was evaluated in the 100 most-cited articles. Each article was counted only once for each country despite the number of variations in addresses. Fifteen articles in the 100 most-cited articles had international collaboration. Among the countries with more than 5 included articles, Romania accounted for the highest proportion of articles with international authors (83.3%) while international collaboration was not implemented in South Korea and Japan. The collaboration between the European countries was the most frequent (n=10). The visualized results of the international collaboration were presented in Figure 3. The circle's size represented the degree of international collaboration of each country and the width of the line linking the countries illustrated the strength of collaboration. France contributed the most articles with international collaboration (n=9) and had collaborated with 6 countries (Italy, Spain, Morocco, China, USA, and Romania). In the international collaboration, France maintained close cooperation in the topic of transient elastography with Italy (link strength=4) and China (link strength=3). There were only 2 different types in the included articles, the ‘original research article' (n=92) and the ‘review' (n=8).

**Figure 4:**
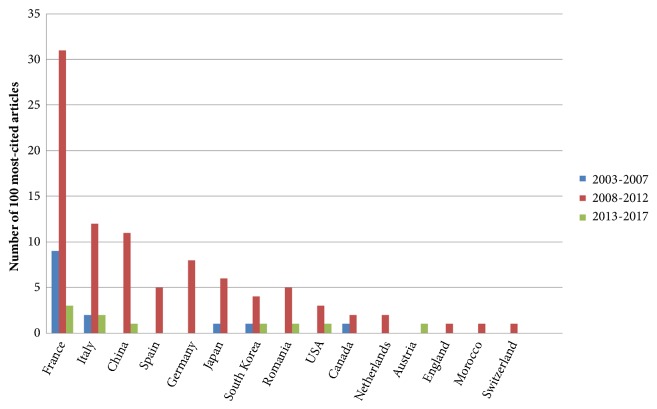
Conditions of transient elastography's research in different countries over time. The number of most-cited articles in each country in three periods which are represented by three colors: the period from 2003 to 2007 (blue), the period from 2008 to 2012 (red), and the period from 2013 to 2017 (green). If no article was on the list of the 100 most-cited articles from a particular country in a certain period, there will be no bar referring to that country shown in the period.

Time span was equally divided into three consecutive periods. During the period from 2003 to 2007, 14 articles had been published and the top-cited one was a “*Prospective Comparison of Transient Elastography, Fibrotest, APRI, and Liver Biopsy for the Assessment of Fibrosis in Chronic Hepatitis C *(n=1380) [18],” published in 2005. In this period, the number of the mean annual citations was 29.71. During the period from 2008 to 2012, 77 articles had been published and the top-cited one was a “*Noninvasive Evaluation of Liver Fibrosis using Transient Elastography* (n=599) [19],” published in 2008. In this period, the number of the mean annual citations was 15.14. During the period from 2013 to 2017, 9 articles had been published and the top-cited one was “*Meta-Analysis: ARFI Elastography versus Transient Elastography for the Evaluation of Liver Fibrosis* (n=159) [20],” published in 2013. In this period, the number of the mean annual citations was 15.50. Numbers of most-cited articles in different countries in three periods were presented in Figure 4. All the three top-cited articles in three periods were contributed by France.

## 4. Discussion

In this study, a bibliometric summary of top-cited articles on transient elastography was presented. It furnishes us with a unique insight into the history and development of transient elastography. The Science Citation Index Expanded (SCI-E) was designated in this study. This database includes over 12,000 of the journals with the highest impact [21], containing open-access journals which were selected by using qualified and rigorous editorial criteria. Also, it has advantages when compared with the others, since the detailed data (e.g., journal sources, annual publications, author, country, and institution) are not included in Medline. Other databases (e.g., Scopus, Google Scholar, PubMed, and Ovid) were not analyzed because SCI-E could provide relatively comprehensive and objective analysis of data [21, 22].

The number of citations was generally applied to appraise the impact of an article and assess the quality of a journal [7]. What is more, the articles with the highest number of citations exert profound influences on the development and research trends of the given field because they provided the foundation for future theory, technology, and application [23]. The list of the most-cited articles would be of interest to several stakeholders for the reasons below. First of all, it reveals numerously utility information about the features of highly cited works such as author, journal, research area, and topic. Secondly, the evolvement of the research field over time would be directly provided for the readers. Last but not least, the characteristics of highly cited articles will help clinicians because it can proclaim important advancements in transient elastography, many of which may be at their early stages of clinical application.

In the 2003-2007 publications, “*Prospective Comparison of Transient Elastography, Fibrotest, APRI, and Liver Biopsy for the Assessment of Fibrosis in Chronic Hepatitis C* [18],” the article with the highest citations focused on assessing the performance of FibroScan in patients with chronic hepatitis C when compared and combined with other available biochemical markers for liver cirrhosis (Fibrotest; Biopredictive; and the aspartate transaminase to platelets ratio index [APRI]). The top-cited article in 2008-2012, “*Noninvasive Evaluation of Liver Fibrosis using Transient Elastography* [19],” introduced the scope of application, advantages, and prospect of transient elastography. The top-cited article in 2012-2017, “*Meta-Analysis: ARFI Elastography versus Transient Elastography for the Evaluation of Liver Fibrosis* [20],” compared the diagnostic performance of acoustic radiation force impulse (ARFI) elastography and transient elastography (TE) of liver fibrosis and demystify that ARFI and TE have parallel diagnostic performance in the detection of significant fibrosis but ARFI is more reliable.

The number of citations of the 100 most-cited articles in the field of transient elastography ranged from 54 to 1376, which were lower than those observed in Imaging Literature, covering a range from 624 to 6447.27. It may be explained by the fact that there were only 19 years since transient elastography had been invented in the world and transient elastography is a specific diagnostic method for liver cirrhosis. When stratified by time, the number of included articles was steadily increasing from 2003 to 2011 except the years 2004 and 2008. The number of included articles began to drop off since 2011.

From the increasing included articles from 2003 to 2011, we can deduce that the research in the field of transient elastography is in a stage of rapid development while the decrease of included articles since 2011 could be ascribed to publication time because the number of citations would increase with the passage of time.

The top three prolific authors are de Ledinghen V., Castera L., and Foucher J. All of them are affiliated to Hop Haut Leveque, CHU Bordeaux, and the collaboration among them is very common. They were principally focusing on the application on liver fibrosis especially in patients with hepatitis C. “*Journal of Hepatology*”, “*Hepatology*”, and “*Liver International*” occupied the first, second, and third most productive journal on transient elastography, respectively. “*Journal of Hepatology*” and “*Hepatology*”, with the impact factor of more than 12, are the leading journals in the topic of hepatology while the impact factor of “*Liver International*” is less than 5. However, the average citations of the included articles in these three journals are more than 120. This may reflect the fact that, in the field of transient elastography, the citations that journals receive are not necessarily correlated with impact factor.

In this study, authors from France contributed 43 articles (8 with international collaboration) in the 100 most-cited articles, ranking the first. This circumstance suggests that France is the leading country and makes a tremendous contribution to the application and popularization of transient elastography. European countries (n=71) made the greatest contribution to the 100 most-cited articles, followed by Asian countries (n=25). But the voice from America (n=7), Africa (n=1), and Oceania (n=1) was weak in this study. Sandrin, a French researcher, was the first one to publish the article about using transient elastography to measure liver stiffness [24]. It is worth mentioning that France and Europe have become the leading country and continent. The study also found that both HBV and HCV could evolve into cirrhosis but they affect disparate area [25, 26]. Sixty-eight percent of people with chronic HBV infection come from Africa and Western Pacific region while Europe and Mediterranean region suffer more from the infection of HCV [27]. The research on transient elastography was focusing on the patients with HBV. The demand of diagnosis and treatment methods promotes popularization of transient elastography in Europe and Asian, especially China because China has the largest population. The international collaboration is more common in European countries (n=15). In contrast, the authors from Asian countries tend to finish the study with the researchers in the motherland (n=3).

There are several deficiencies in this study. First, self-citation, citations in textbooks, and lectures were not included in this study and the authors were more likely to cite the articles published in the journal in which they sought to publish their work subsequently [28, 29]. Besides, the publication time played an apparent role in citations analysis since the number of citations would increase over time [30–32]. Moreover, the SCI-EXPANDED has an extensive coverage of English-language journals but one of its limitations is that publications in non-English are only a few [21, 22]. There was an obvious bias for articles published in English journals and articles published in non-English journals were clearly at a disadvantage in the citation analysis.

## 5. Conclusions

We identified and analyzed the 100 most-cited articles focusing on transient elastography. The research of transient elastography is in rapid development. Transient elastography has become a mature technology for evaluating liver fibrosis. The combination of transient elastography with other noninvasive assessment of liver fibrosis could be a more accurate evaluation of liver fibrosis in the future.

## Figures and Tables

**Table 1:** Journals with more than three articles in the 100 most-cited articles.

Journal	Number of articles	IF in 2017
Journal of Hepatology	20	12.486
Hepatology	14	13.246
Liver International	7	4.116
Alimentary Pharmacology & Therapeutics	6	7.286
Journal of Viral Hepatitis	6	4.122
Gastroenterology	4	18.392
Journal of Gastroenterology	4	4.658
American Journal of Gastroenterology	3	9.566
Digestive and Liver Disease	3	3.061
Journal of Gastroenterology and Hepatology	3	3.452

**Table 2:** The authors with more than five articles in the 100 most-cited articles on transient elastography.

Author	Number of articles	First author	corresponding author
		(number of articles)	
de Ledinghen, V	24	7	14
Castera, L	17	8	8
Foucher, J	15	2	0
Vergniol, J	12	1	0
Merrouche, W	10	0	0
Le Bail, B	10	0	0
Chan, HLY	9	1	5
Wong, GLH	9	5	0
Wong, VWS	9	2	1
Couzigou, P	8	0	0
Beaugrand, M	7	0	0
Choi, PCL	7	0	0
Chan, AWH	6	0	0
Han, KH	5	0	2
Kim, DY	5	1	1
Ahn, SH	5	0	0
Bernard, PH	5	0	0
Chim, AML	5	0	0
Chon, CY	5	0	0
Park, JY	5	0	0
Yiu, KKL	5	0	0
Ziol, M	5	0	0

## Data Availability

All additional data were attached to this system.

## Supplementary Materials

Supplementary Materials Supplementary Table 1. The 100 most-cited articles in the field of transient elastography ranked in order of the number of citations received. Rank A is the ranking of total citations; Rank B is the ranking of annual citations.
