# Supplementary material for: Emerging Trends and New Developments in Transient Elastography: A Bibliometric and Cocitation Analysis from 1999 to 2017
Source: Can J Gastroenterol Hepatol. 2019 Mar 5;2019:3280657. doi: 10.1155/2019/3280657 (PMC6425420; doi:10.1155/2019/3280657)
Supplement: Supplementary Materials — Supplementary Table 1. The 100 most-cited articles in the field of transient elastography ranked in order of the number of citations received. Rank A is the ranking of total citations; Rank B is the ranking of annual citations. [file 3280657.f1.docx]

| Supplementary Table 1  The 100 most-cited articles in the field of Transient elastography ranked in order of the number of citations received. | | | |
| --- | --- | --- | --- |
| Rank A | Article | Number of citations | Number of citations/year (Rank B) |
| 1 | Castéra L, Vergniol J, Foucher J, et al. Prospective comparison of transient elastography, Fibrotest, APRI, and liver biopsy for the assessment of fibrosis in chronic hepatitis C.[J]. Gastroenterology, 2005, 128(2):343-350. | 1376 | 98.29 （1） |
| 2 | Sandrin L, Fourquet B, Hasquenoph J M, et al. Transient elastography: a new noninvasive method for assessment of hepatic fibrosis.[J]. Ultrasound in Medicine & Biology, 2003, 29(12):1705-1713. | 1333 | 83.31 （2） |
| 3 | Foucher J, Chanteloup E, Vergniol J, et al. Diagnosis of cirrhosis by transient elastography (FibroScan): a prospective study[J]. Digest of the World Core Medical Journals(Gastroenterology), 2006, 55(3):403. | 713 | 54.85 （5） |
| 4 | Castera L, Forns X, Alberti A. Non-invasive evaluation of liver fibrosis using transient elastography.[J]. Journal of Hepatology, 2008, 48(5):835-847. | 598 | 54.36 （6） |
| 5 | Castéra L, Foucher J, Bernard P H, et al. Pitfalls of liver stiffness measurement: a 5-year prospective study of 13,369 examinations.[J]. Hepatology, 2010, 51(3):828-835. | 502 | 55.78 （4） |
| 6 | Musso G, Gambino R, Cassader M, et al. Meta-analysis: Natural history of non-alcoholic fatty liver disease (NAFLD) and diagnostic accuracy of non-invasive tests for liver disease severity[J]. Annals of Medicine, 2011, 43(8):617-49. | 450 | 56.25 （3） |
| 7 | Wong V W, Vergniol J, Wong G L, et al. Diagnosis of fibrosis and cirrhosis using liver stiffness measurement in nonalcoholic fatty liver disease.[J]. Hepatology, 2010, 51(2):454-462. | 425 | 47.22 （7） |
| 8 | Coco B, Oliveri F, Maina A M, et al. Transient elastography: a new surrogate marker of liver fibrosis influenced by major changes of transaminases[J]. Journal of Viral Hepatitis, 2007, 14(5):360–369. | 408 | 34 （10） |
| 9 | Gannecarrié N, Ziol M, De L V, et al. Accuracy of liver stiffness measurement for the diagnosis of cirrhosis in patients with chronic liver diseases.[J]. Hepatology, 2006, 44(6):1511-1517. | 332 | 25.54 （17） |
| 10 | Song Z Z. Re: Transient elastography is unreliable for detection of cirrhosis in patients with acute liver damage.[J]. Hepatology, 2008, 48(4):1351–1352. | 309 | 28.09  （14） |
| 11 | Marcellin P, Ziol M, Bedossa P, et al. Non‐invasive assessment of liver fibrosis by stiffness measurement in patients with chronic hepatitis B[J]. Liver International, 2009, 29(2):242-247. | 301 | 30.1 （11） |
| 12 | Millonig G, Reimann F M, Friedrich S, et al. Extrahepatic cholestasis increases liver stiffness (FibroScan) irrespective of fibrosis[J]. Hepatology, 2008, 48(5):1718-1723. | 290 | 26.36（16） |
| 13 | De L V, Douvin C, Kettaneh A, et al. Diagnosis of hepatic fibrosis and cirrhosis by transient elastography in HIV/hepatitis C virus-coinfected patients.[J]. J Acquir Immune Defic Syndr, 2006, 41(2):175-179. | 262 | 20.15 (26) |
| 14 | Roulot D, Czernichow S, Le C H, et al. Liver stiffness values in apparently healthy subjects: influence of gender and metabolic syndrome[J]. Journal of Hepatology, 2008, 48(4):606-613. | 251 | 22.82 (22) |
| 15 | Castera L. Noninvasive methods to assess liver disease in patients with hepatitis B or C.[J]. Gastroenterology, 2012, 142(6):1293-1302. | 250 | 35.71 （8） |
| 16 | Ferraioli G, Tinelli C, Bello B D, et al. Accuracy of real‐time shear wave elastography for assessing liver fibrosis in chronic hepatitis C: A pilot study[J]. Hepatology, 2012, 56(6):2125-2133. | 247 | 35.29 （9） |
| 17 | Chan H, Wong G P, Chan A, et al. Alanine aminotransferase-based algorithms of liver stiffness measurement by transient elastography (Fibroscan) for liver fibrosis in chronic hepatitis B[J]. Journal of Viral Hepatitis, 2009, 16(1):36-44. | 243 | 24.3 （19） |
| 18 | Kazemi F, Kettaneh A, N'Kontchou G, et al. Liver stiffness measurement selects patients with cirrhosis at risk of bearing large oesophageal varices[J]. Journal of Hepatology, 2006, 45(2):230. | 239 | 18.38 (31) |
| 19 | Bavu E, Gennisson J L, Couade M, et al. Noninvasive in vivo liver fibrosis evaluation using supersonic shear imaging: a clinical study on 113 hepatitis C virus patients.[J]. Ultrasound in Medicine & Biology, 2011, 37(9):1361-1373. | 225 | 28.13 （13） |
| 20 | Foucher J, Castéra L, Bernard P H, et al. Prevalence and factors associated with failure of liver stiffness measurement using FibroScan in a prospective study of 2114 examinations.[J]. Eur J Gastroenterol Hepatol, 2006, 18(4):411-412. | 223 | 17.15 (34) |
| 21 | Castéra L, Bail B L, Roudot F. Early detection in routine clinical practice of cirrhosis and oesophageal varices in chronic hepatitis C: comparison of transient elastography (FibroScan) with standard laboratory tests and non-invasive scores.[J]. Journal of Hepatology, 2009, 50(1):59-68. | 220 | 22 (24) |
| 22 | Millonig G, Friedrich S, Adolf S, et al. Liver stiffness is directly influenced by central venous pressure[J]. Journal of Hepatology, 2010, 52(2):206-10. | 216 | 24 (20) |
| 23 | Shaheen A A M, Wan A F, Myers R P. FibroTest and FibroScan for the Prediction of Hepatitis C-Related Fibrosis: A Systematic Review of Diagnostic Test Accuracy[J]. American Journal of Gastroenterology, 2007, 102(11):2589. | 216 | 18 (32) |
| 24 | Yoneda M, Yoneda M, Mawatari H, et al. Noninvasive assessment of liver fibrosis by measurement of stiffness in patients with nonalcoholic fatty liver disease (NAFLD)[J]. Digestive & Liver Disease Official Journal of the Italian Society of Gastroenterology & the Italian Association for the Study of the Liver, 2008, 40(5):371-8. | 210 | 19.09 (29) |
| 25 | Myers R P, Pomier-Layrargues G, Kirsch R, et al. Feasibility and diagnostic performance of the FibroScan XL probe for liver stiffness measurement in overweight and obese patients[J]. Hepatology, 2012, 55(1):199-208. | 209 | 29.86 （12） |
| 26 | Degos F, Perez P, Roche B, et al. Diagnostic accuracy of FibroScan and comparison to liver fibrosis biomarkers in chronic viral hepatitis: a multicenter prospective study (the FIBROSTIC study).[J]. Journal of Hepatology, 2010, 53(6):1013-1021. | 206 | 22.89 (21) |
| 27 | Nguyen D, Talwalkar J A. Noninvasive assessment of liver fibrosis[J]. Hepatology, 2005, 53(6):667-683. | 200 | 25 （18） |
| 28 | Bureau C, Metivier S, Peron J M, et al. Transient elastography accurately predicts presence of significant portal hypertension in patients with chronic liver disease[J]. Alimentary Pharmacology & Therapeutics, 2008, 27(12):1261-1268. | 172 | 15.64 (36) |
| 29 | Jung K S, Kim S U, Ahn S H, et al. Risk Assessment of Hepatitis B Virus-Related Hepatocellular Carcinoma Development Using Liver Stiffness Measurement (FibroScan)[J]. Hepatology, 2011, 53(3):885-94. | 170 | 21.25 (25) |
| 30 | Kettaneh A, Marcellin P, Douvin C, et al. Features associated with success rate and performance of FibroScan measurements for the diagnosis of cirrhosis in HCV patients: a prospective study of 935 patients[J]. Journal of Hepatology, 2007, 46(4):628-34. | 170 | 14.17 (40) |
| 31 | Bota S, Herkner H, Sporea I, et al. Meta-analysis: ARFI elastography versus transient elastography for the evaluation of liver fibrosis[J]. Liver International Official Journal of the International Association for the Study of the Liver, 2013, 33(8):1138–1147. | 159 | 26.5 （15） |
| 32 | Colecchia A; Montrone L; Scaioli E; Bacchi-Reggiani ML; Colli A; Casazza G; Schiumerini R; Turco L; Di Biase AR; Mazzella G; Marzi L; Arena U; Pinzani M; Festi D. Exam 2: Measurement of Spleen Stiffness to Evaluate Portal Hypertension and the Presence of Esophageal Varices in Patients With HCV-Related Cirrhosis[J]. Gastroenterology, 2012, 143(3):646. | 158 | 22.57 (23) |
| 33 | Colletta C, Smirne C, Fabris C, et al. Value of two noninvasive methods to detect progression of fibrosis among HCV carriers with normal aminotransferases.[J]. Hepatology, 2005, 42(4):838-45. | 151 | 10.79 (54) |
| 34 | Castera L, Pinzani M, Bosch J. Non invasive evaluation of portal hypertension using transient elastography[J]. Journal of Hepatology, 2012, 56(3):696-703. | 136 | 19.43 (28) |
| 35 | Lucidarme D, Foucher J, Bail B L, et al. Factors of accuracy of transient elastography (fibroscan) for the diagnosis of liver fibrosis in chronic hepatitis C[J]. Hepatology, 2009, 49(4):1083-1089. | 135 | 13.5 (42) |
| 36 | Robic MA; Procopet B; Métivier S; Péron JM; Selves J; Vinel JP; Bureau C. Liver stiffness accurately predicts portal hypertension related complications in patients with chronic liver disease: a prospective study.[J]. Journal of Hepatology, 2011, 55(5):1017. | 134 | 16.75 (35) |
| 37 | Lédinghen V D, Vergniol J, Foucher J, et al. Non-invasive diagnosis of liver steatosis using controlled attenuation parameter (CAP) and transient elastography[J]. Liver International Official Journal of the International Association for the Study of the Liver, 2012, 32(6):911. | 122 | 17.43 (33) |
| 38 | Baranova A, Lal P, Birerdinc A, et al. Non-Invasive markers for hepatic fibrosis[J]. Bmc Gastroenterology, 2011, 11(1):91. | 119 | 14.88 (38) |
| 39 | Gaia S, Carenzi S, Barilli A L, et al. Reliability of transient elastography for the detection of fibrosis in non-alcoholic fatty liver disease and chronic viral hepatitis[J]. Journal of Hepatology, 2011, 54(1):64-71. | 114 | 14.25 (39) |
| 40 | Castéra L, Sebastiani G, Bail B L, et al. Prospective comparison of two algorithms combining non-invasive methods for staging liver fibrosis in chronic hepatitis C ☆[J]. Journal of Hepatology, 2010, 52(2):191-198. | 114 | 12.67 (46) |
| 41 | De L V, Le B B, Rebouissoux L, et al. Liver stiffness measurement in children using FibroScan: feasibility study and comparison with Fibrotest, aspartate transaminase to platelets ratio index, and liver biopsy[J]. Journal of Pediatric Gastroenterology & Nutrition, 2007, 45(4):443. | 111 | 9.25 (59) |
| 42 | Wong V W, Vergniol J, Wong G L, et al. Liver stiffness measurement using XL probe in patients with nonalcoholic fatty liver disease.[J]. American Journal of Gastroenterology, 2012, 107(12):1862-71. | 109 | 15.57 (37) |
| 43 | Sebastiani G, Halfon P, Castera L, et al. SAFE biopsy: a validated method for large-scale staging of liver fibrosis in chronic hepatitis C.[J]. Hepatology, 2009, 49(6):1821–1827. | 105 | 10.5 (55) |
| 44 | De L V, Vergniol J, Foucher J, et al. Feasibility of liver transient elastography with FibroScan using a new probe for obese patients[J]. Liver International Official Journal of the International Association for the Study of the Liver, 2010, 30(7):1043-8. | 102 | 11.33 (52) |
| 45 | Wong GL; Wong VW; Choi PC; Chan AW; Chum RH; Chan HK; Lau KK; Chim AM; Yiu KK; Chan FK; Sung JJ; Chan HL. Assessment of fibrosis by transient elastography compared with liver biopsy and morphometry in chronic liver diseases.[J]. Clin Gastroenterol Hepatol, 2008, 6(9):1027-1035. | 101 | 9.18 (61) |
| 46 | Nguyenkhac E, Chatelain D, Tramier B, et al. Assessment of asymptomatic liver fibrosis in alcoholic patients using fibroscan: prospective comparison with seven non-invasive laboratory tests.[J]. Alimentary Pharmacology & Therapeutics, 2008, 28(10):1188. | 100 | 9.09 (63) |
| 47 | Cassinotto C, De L V. Non-invasive assessment of liver fibrosis with impulse elastography: Comparison of Supersonic Shear Imaging with ARFI and FibroScan[J]. Journal of Hepatology, 2015, 61(3):550-557. | 99 | 19.8 (27) |
| 48 | Friedrich-Rust M; Romen D; Vermehren J; Kriener S; Sadet D; Herrmann E; Zeuzem S; Bojunga J. Acoustic radiation force impulse-imaging and transient elastography for non-invasive assessment of liver fibrosis and steatosis in NAFLD[J]. European Journal of Radiology, 2012, 81(3):e325. | 92 | 13.14 (43) |
| 49 | Lédinghen V D, Wong W S, Vergniol J, et al. Diagnosis of liver fibrosis and cirrhosis using liver stiffness measurement: Comparison between M and XL probe of FibroScan®;[J]. Journal of Hepatology, 2012, 56(4):833-839. | 90 | 12.86 (44) |
| 50 | Smith J O, Sterling R K. Systematic review: non‐invasive methods of fibrosis analysis in chronic hepatitis C[J]. Alimentary Pharmacology & Therapeutics, 2009, 30(6):557–576. | 90 | 9 (64) |
| 51 | Zarski J P, Sturm N, Guechot J, et al. Comparison of nine blood tests and transient elastography for liver fibrosis in chronic hepatitis C: The ANRS HCEP-23 study[J]. Journal of Hepatology, 2012, 56(1):55-62. | 89 | 12.71 (45) |
| 52 | Ogawa E, Furusyo N, Toyoda K, et al. The longitudinal quantitative assessment by transient elastography of chronic hepatitis C patients treated with pegylated interferon alpha-2b and ribavirin.[J]. Antiviral Res, 2009, 83(2):127-134. | 89 | 8.9 (67) |
| 53 | Vergniol J, Foucher J, Castéra L, et al. Changes of non-invasive markers and FibroScan values during HCV treatment[J]. Journal of Viral Hepatitis, 2009, 16(2):132-140. | 89 | 8.9 (68) |
| 54 | Crespo G, Fernándezvaro G, Mariño Z, et al. ARFI, FibroScan, ELF, and their combinations in the assessment of liver fibrosis: a prospective study.[J]. Journal of Hepatology, 2012, 57(2):281-287. | 87 | 12.43 (48) |
| 55 | LupåŸOr M, Badea R, StefäƒNescu H, et al. Analysis of histopathological changes that influence liver stiffness in chronic hepatitis C. Results from a cohort of 324 patients[J]. J Gastrointestin Liver Dis, 2008, 17(2):155-163. | 87 | 7.91 (74) |
| 56 | Oliveri F, Coco B, Ciccorossi P, et al. Liver stiffness in the hepatitis B virus carrier:A non-invasive marker of liver disease influenced by the pattern of transaminases[J]. World Journal of Gastroenterology, 2008, 14(40):6154-6162. | 86 | 7.82 (76) |
| 57 | Festi D, Schiumerini R, Marzi L, et al. Review article: the diagnosis of non-alcoholic fatty liver disease -- availability and accuracy of non-invasive methods[J]. Alimentary Pharmacology & Therapeutics, 2013, 37(4):392. | 84 | 14 (41) |
| 58 | Mueller S, Millonig G, Sarovska L, et al. Increased liver stiffness in alcoholic liver disease: differentiating fibrosis from steatohepatitis.[J]. World Journal of Gastroenterology, 2010, 16(8):966-972. | 82 | 9.11 (62) |
| 59 | Boursier J, Isselin G, Fouchard-Hubert I, et al. Acoustic radiation force impulse: a new ultrasonographic technology for the widespread noninvasive diagnosis of liver fibrosis[J]. European Journal of Gastroenterology & Hepatology, 2010, 22(9):1074. | 81 | 9 (65) |
| 60 | Rifai K, Cornberg J, Mederacke I, et al. Clinical feasibility of liver elastography by acoustic radiation force impulse imaging (ARFI)[J]. Digestive & Liver Disease Official Journal of the Italian Society of Gastroenterology & the Italian Association for the Study of the Liver, 2011, 43(6):491. | 80 | 10 (56) |
| 61 | Kim B K, Han K H, Park J Y, et al. A Liver Stiffness Measurement-Based, Noninvasive Prediction Model for High-Risk Esophageal Varices in B-Viral Liver Cirrhosis[J]. American Journal of Gastroenterology, 2010, 105(6):1382-1390. | 80 | 8.89 (69) |
| 62 | Kim K M, Choi W B, Park S H, et al. Diagnosis of hepatic steatosis and fibrosis by transient elastography in asymptomatic healthy individuals: a prospective study of living related potential liver donors[J]. Journal of Gastroenterology, 2007, 42(5):382-388. | 78 | 6.5 (89) |
| 63 | Stefanescu H, Grigorescu M, Lupsor M, et al. Spleen stiffness measurement using Fibroscan for the noninvasive assessment of esophageal varices in liver cirrhosis patients.[J]. Journal of Gastroenterology & Hepatology, 2011, 26(1):164. | 77 | 9.63 (58) |
| 64 | Liver E A F S O. EASL-ALEH Clinical Practice Guidelines: Non-invasive tests for evaluation of liver disease severity and prognosis[J]. Journal of Hepatology, 2015, 63(1):237-264. | 76 | 19 (30) |
| 65 | Kim D Y, Kim S U, Ahn S H, et al. Usefulness of FibroScan for detection of early compensated liver cirrhosis in chronic hepatitis B[J]. Digestive Diseases & Sciences, 2009, 54(8):1758-1763. | 76 | 7.6 (81) |
| 66 | Poynard T, Munteanu M, Luckina E, et al. Liver fibrosis evaluation using real-time shear wave elastography: applicability and diagnostic performance using methods without a gold standard.[J]. Journal of Hepatology, 2013, 58(5):928-35. | 75 | 12.5 (47) |
| 67 | Thabut D, Moreau R, Lebrec D. Noninvasive assessment of portal hypertension in patients with cirrhosis[J]. Hepatology, 2011, 53(2):683-94. | 74 | 9.25 (60) |
| 68 | Boursier J, Konaté A, Gorea G, et al. Reproducibility of Liver Stiffness Measurement by Ultrasonographic Elastometry[J]. Clinical Gastroenterology & Hepatology, 2008, 6(11):1263-1269. | 73 | 6.64 (86) |
| 69 | Shi K Q, Fan Y C, Pan Z Z, et al. Transient elastography: a meta-analysis of diagnostic accuracy in evaluation of portal hypertension in chronic liver disease[J]. Liver International, 2013, 33(1):62–71. | 72 | 12 (51) |
| 70 | Piscaglia F, Salvatore V, Di D R, et al. Accuracy of VirtualTouch Acoustic Radiation Force Impulse (ARFI) imaging for the diagnosis of cirrhosis during liver ultrasonography[J]. Ultraschall in Der Medizin, 2011, 32(2):167-175. | 72 | 9 (66) |
| 71 | Friedrichrust M, Rosenberg W, Parkes J, et al. Comparison of ELF, FibroTest and FibroScan for the non-invasive assessment of liver fibrosis[J]. Bmc Gastroenterology, 2010, 10(1):1-8. | 72 | 8 (72) |
| 72 | Myers R P, Pomier-Layrargues G, Kirsch R, et al. Discordance in fibrosis staging between liver biopsy and transient elastography using the FibroScan XL probe.[J]. Journal of Hepatology, 2012, 56(3):564-70. | 70 | 10 (57) |
| 73 | Wong G L H, Wong V W S, Choi P C L, et al. Development of a non‐invasive algorithm with transient elastography (Fibroscan) and serum test formula for advanced liver fibrosis in chronic hepatitis B[J]. Aliment Pharmacol Ther, 2010, 31(10):1095-1103. | 70 | 7.78 (77) |
| 74 | Castellares C, Barreiro P, Martín-Carbonero L, et al. Liver cirrhosis in HIV‐infected patients: prevalence, aetiology and clinical outcome[J]. Journal of Viral Hepatitis, 2008, 15(3):165. | 70 | 6.36 (91) |
| 75 | Sánchez-Conde M, Montes-Ramírez M L, Miralles P, et al. Comparison of transient elastography and liver biopsy for the assessment of liver fibrosis in HIV/hepatitis C virus-coinfected patients and correlation with noninvasive serum markers[J]. J Viral Hepat, 2010, 17(4):280-286. | 69 | 7.67 (79) |
| 76 | Kirk G D, Mehta S H, Astemborski J, et al. HIV, Age, and the Severity of Hepatitis C Virus–Related Liver Disease: A Cohort Study[J]. Annals of Internal Medicine, 2013, 158(9):658. | 68 | 11.33 (53) |
| 77 | Ogawa E, Furusyo N, Toyoda K, et al. Transient elastography for patients with chronic hepatitis B and C virus infection: Non-invasive, quantitative assessment of liver fibrosis[J]. Hepatology Research, 2007, 37(12):1002–1010. | 67 | 5.58 (98) |
| 78 | Mirella F, Cristina R, Giovanni C, et al. Etiology-related determinants of liver stiffness values in chronic viral hepatitis B or C[J]. Journal of Hepatology, 2011, 54(4):621-628. | 66 | 8.25 (71) |
| 79 | Boursier J, Vergniol J, Sawadogo A, et al. The combination of a blood test and Fibroscan improves the non-invasive diagnosis of liver fibrosis[J]. Liver International, 2009, 29(10):1507–1515. | 66 | 6.6 (87) |
| 80 | Harada N, Soejima Y, Taketomi A, et al. Assessment of graft fibrosis by transient elastography in patients with recurrent hepatitis C after living donor liver transplantation.[J]. Transplantation, 2008, 86(5):749; author reply 749. | 66 | 6 (93) |
| 81 | Wong G L, Wong V W, Choi P C, et al. Increased liver stiffness measurement by transient elastography in severe acute exacerbation of chronic hepatitis B[J]. Journal of Gastroenterology & Hepatology, 2009, 24(6):1002-1007. | 65 | 6.5 (90) |
| 82 | Lédinghen V D, Vergniol J. Transient elastography (FibroScan)[J]. Gastroentérologie Clinique Et Biologique, 2008, 32(6):58-67. | 65 | 5.91 (94) |
| 83 | Maida I, Garciagasco P, Sotgiu G, et al. Antiretroviral-associated portal hypertension: a new clinical condition? Prevalence, predictors and outcome.[J]. Antiviral Therapy, 2008, 13(1):103-107. | 63 | 5.73 (96) |
| 84 | Tatsumi C, Kudo M, Ueshima K, et al. Noninvasive evaluation of hepatic fibrosis using serum fibrotic markers, transient elastography (FibroScan) and real-time tissue elastography[J]. Intervirology, 2008, 51(Suppl.1):27-33. | 63 | 5.73 (97) |
| 85 | Ebinuma H, Saito H, Komuta M, et al. Evaluation of liver fibrosis by transient elastography using acoustic radiation force impulse: comparison with Fibroscan(Â®)[J]. Journal of Gastroenterology, 2011, 46(10):1238-1248. | 62 | 7.75 (78) |
| 86 | Corpechot C, Gaouar F, El N A, et al. Baseline values and changes in liver stiffness measured by transient elastography are associated with severity of fibrosis and outcomes of patients with primary sclerosing cholangitis[J]. Gastroenterology, 2014, 146(4):970. | 61 | 12.2 (49) |
| 87 | Wong G L, Chan H L, Wong C K, et al. Liver stiffness-based optimization of hepatocellular carcinoma risk score in patients with chronic hepatitis B.[J]. Journal of Hepatology, 2014, 60(2):339-345. | 61 | 12.2 (50) |
| 88 | Boursier J, De L V, Zarski J P, et al. Comparison of eight diagnostic algorithms for liver fibrosis in hepatitis C: new algorithms are more precise and entirely noninvasive † ‡[J]. Hepatology, 2012, 55(1):58. | 61 | 8.71 (70) |
| 89 | Hã©Zode C, Castã©Ra L, Roudot-Thoraval F, et al. Liver stiffness diminishes with antiviral response in chronic hepatitis C[J]. Alimentary Pharmacology & Therapeutics, 2011, 34(6):656-663. | 61 | 7.63 (80) |
| 90 | Wang J H, Changchien C S, Hung C H, et al. FibroScan and ultrasonography in the prediction of hepatic fibrosis in patients with chronic viral hepatitis[J]. Journal of Gastroenterology, 2009, 44(5):439-446. | 61 | 6.1 (92) |
| 91 | Castera L. Invasive and non-invasive methods for the assessment of fibrosis and disease progression in chronic liver disease[J]. Best Practice & Research Clinical Gastroenterology, 2011, 25(2):291-303. | 60 | 7.5 (82) |
| 92 | Wong G L, Wong V W, Chim A M, et al. Factors associated with unreliable liver stiffness measurement and its failure with transient elastography in the Chinese population[J]. Journal of Gastroenterology & Hepatology, 2011, 26(2):300-305. | 60 | 7.5 (83) |
| 93 | Lupsor M; Badea R; Stefanescu H; Grigorescu M; Serban A; Radu C; Crişan D; Sparchez Z; Iancu S; Maniu A. Performance of unidimensional transient elastography in staging non-alcoholic steatohepatitis.[J]. J Gastrointestin Liver Dis, 2010, 19(1):53-60. | 60 | 6.67 (85) |
| 94 | Arndt R, Schmidt S, Loddenkemper C, et al. Noninvasive evaluation of renal allograft fibrosis by transient elastography--a pilot study.[J]. Transplant International Official Journal of the European Society for Organ Transplantation, 2010, 23(9):871-877. | 59 | 6.56 (88) |
| 95 | Corradi F, Piscaglia F, Flori S, et al. Assessment of liver fibrosis in transplant recipients with recurrent HCV infection: Usefulness of transient elastography[J]. Digestive & Liver Disease Official Journal of the Italian Society of Gastroenterology & the Italian Association for the Study of the Liver, 2009, 41(3):217. | 59 | 5.9 (95) |
| 96 | De-Ledinghen V, Barreiro P, Foucher J, et al. Liver fibrosis on account of chronic hepatitis C is more severe in HIV-positive than HIV-negative patients despite antiretroviral therapy.[J]. J Viral Hepat, 2008, 15(6):427–433. | 57 | 5.18 (99) |
| 97 | Yoon K T. Liver Stiffness Measurement Using Acoustic Radiation Force Impulse (ARFI) Elastography and Effect of Necroinflammation[J]. Digestive Diseases & Sciences, 2012, 57(6):1682-1691. | 56 | 8 (73) |
| 98 | Up K S, Hoon L J, Young K D, et al. Prediction of Liver-Related Events Using Fibroscan in Chronic Hepatitis B Patients Showing Advanced Liver Fibrosis[J]. Plos One, 2012, 7(5):e36676. | 55 | 7.86 (75) |
| 99 | Morikawa H, Fukuda K, Kobayashi S, et al. Real-time tissue elastography as a tool for the noninvasive assessment of liver stiffness in patients with chronic hepatitis C.[J]. Journal of Gastroenterology, 2011, 46(3):350-358. | 55 | 6.88 (84) |
| 100 | Fung J, Lai C L, Fong D Y, et al. Correlation of liver biochemistry with liver stiffness in chronic hepatitis B and development of a predictive model for liver fibrosis[J]. Liver International, 2008, 28(10):1408-1416. | 54 | 4.91 (100) |
